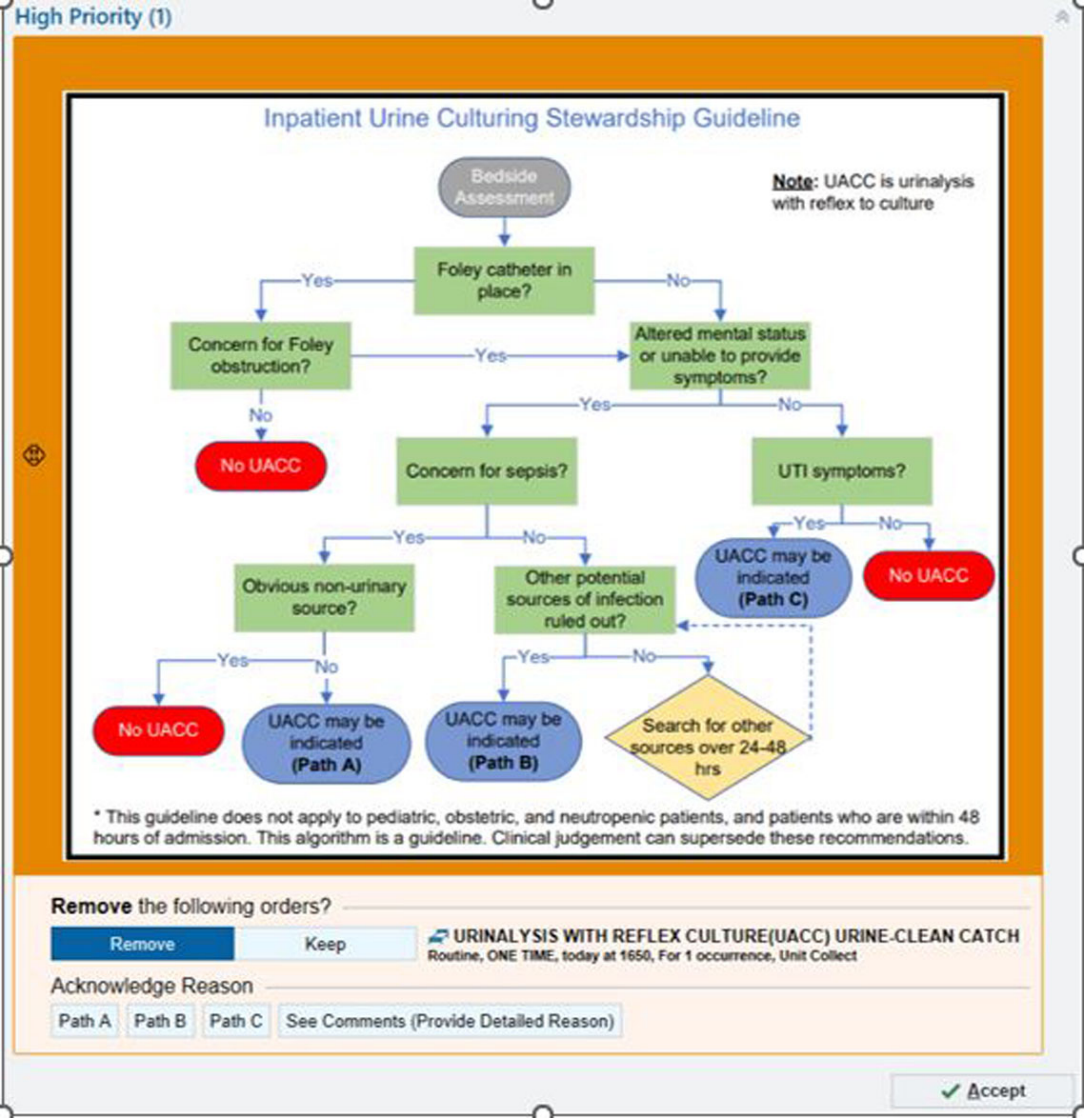# It Takes a Village: Leveraging a Multidisciplinary Team and Technology for Urine Culturing Stewardship

**DOI:** 10.1017/ash.2024.217

**Published:** 2024-09-16

**Authors:** Mandy Swann, Amy Lucas, Christian Ostrowski, Carla Bapst, Lauren Fargis, Robin Strachman, Kathleen Manchin, Maribeth Greenway, Jacob Gillen, Anthony Baffoe-Bonnie

**Affiliations:** Carilion Clinic; Virginia Tech Carilion School of Medicine

## Abstract

**Background:** Patients without urinary tract infection (UTI) symptoms but with a positive urine culture are considered to have asymptomatic bacteriuria (ASB). This often represents colonization and treatment is not recommended or clinically beneficial. Treatment of ASB can promote antimicrobial resistance and increased rates of Clostriodies difficile infections. Many cases of ASB are incorrectly assigned as CAUTIs due to over-culturing practices. We hypothesized that a urine culture algorithm, embedded within a best practice alert (BPA) in the electronic medical record (EMR), would reduce urine culturing practices for ASB. **Methods:** From Feb 2022 through May 2023, a multidisciplinary team implemented an Inpatient Urine Culturing Stewardship Guideline. A BPA fired when a provider placed a urinalysis with reflex to culture (UACC) or urine culture (UC) order for patients who met criteria (Image 1). The BPA directed providers to remove the order, select the appropriate pathway from the guideline, or provide a rationale for placing the order. The intervention was piloted on three intensive care units and two progressive care units, containing both medical and surgical patients. Monthly ordering practices, CAUTI rates, and gram-negative rod (GNR) bacteremia rates from a 13-month pre-intervention baseline period were compared to a 16-month intervention period. Over the same time periods, we also assessed changes in ordering practices for comparison units which did not implement the intervention. Pre-and-post intervention cohorts were analyzed using median two sample tests and Exact Poison Method, as appropriate. **Results:** On intervention units there was a 41.0% reduction in the median number of UACC and UC orders per 1000 patient days from 16.31 during the baseline period to 9.62 in the intervention period (p=0.0036). Pan cultures per 1000 patient days in which one of the orders was a UACC or UC fell by 42.2% from a median of 10.20 per 1000 patient days to 5.90 (p=0.0008). The comparison units saw no significant reductions in UACC and UC orders (p=0.21) or pan cultures (p=1.0). On the intervention units, the CAUTI rate for the baseline period was 1.31 per 1000 catheter days versus 0.79 in the intervention period (IRR = 1.65; p=0.44). GNR bacteremias remained stable on the intervention units between the baseline and intervention periods (p=0.82). **Conclusion:** This multidisciplinary intervention, leveraging EMR clinical decision support, reduced urine and pan culturing practices while demonstrating a trend towards a reduced CAUTI rate. The prevalence of GNR bacteremias remained consistent with baseline levels, suggesting the intervention did not cause harm.

**Figure:**